# Universal brace simulation platform

**DOI:** 10.1186/1748-7161-9-S1-O55

**Published:** 2014-12-04

**Authors:** Grzegorz Śliwiński, Ralf Zeckay, Hagen Malberg, Helmut Diers, Zbigniew Śliwiński, Michael Werner, Jessica Rietze

**Affiliations:** 1 Institute for Biomedical Engineering, Technical University Dresden, Dresden, Germany; 2 Research and Development, Schlangenbad, Germany; 3 Clinic of Rehabilitation WS ZOZ, Zgorzelec, Poland; 4 Group Medical Engineering, Fraunhofer IWU, Dresden, Germany

## Background

According to reports of the German Public Health Department, up to 80% of the children suffer on postural weakness. Most of them develop during the adolescent growth severe spine diseases like scoliosis. In case of slight postural deformity, patients are able to influence actively the posture of the spine by physiotherapy and training of stance and behaviour [1]. In advanced cases the patient cannot actively correct the stance and requires passive supportive measures [2], e.g. braces. Unfortunately the quality of braces and brace therapy results are varying very much due to not standardized procedures and different level of qualification of orthopaedic technicians.

## Aim

The correction principle of brace therapy is based on the reversal of the curvature, extension and de-rotation. The patient’s spine gets strained mechanically by different points of pressure. The most important step in brace treatment is the correct adaption to the patient body. At present mostly an individual plaster cast of the partially-corrected patient is made as basis for brace adaption. It is the aim of the study to develop a universal platform for simulation of brace construction.

## Design

Initial studies in 2009 by Helmut Diers and Asklepios Clinic Bad Abbach show that it should be possible to use the three-dimensional setting of the FED-compression-system [3] as a simulation platform for simulation of the construction of braces. In addition a radiation free 3D Spine and Surface Topography system will be an integrated part of the technical design.

## Methods

Based on existing experience and the construction and function of a FED system it will be possible to visualize immediately the effect of the correction forces on the spine curvature so that, if necessary, new force transmission points can be chosen. In this way the efficiency of the brace treatment can be monitored, changes are easily possible and results will be improved. To visualize the brace effect, a spine measurement system is integrated the spine curvatures and enables the optimization of the brace-referred adaption parameters

## Results

It is expected that the research results will improve the patient-individual brace-care. Using the existing FED therapeutic device for scoliosis treatment and integration of surface topography, it will be possible to develop a simulation platform for visualizing the forces in brace treatment. In this way the medical benefit will be considerably improved as well as the quality and economic efficiency of brace production.

